# Intimate partner violence types are differentially associated with substance use among young, urban, sexual minority men of color

**DOI:** 10.1371/journal.pone.0309958

**Published:** 2024-09-06

**Authors:** Joshua A. Rusow, Ankur Srivastava, Bethany C. Bray, Jeremy T. Goldbach, Michele D. Kipke

**Affiliations:** 1 Brown School of Social Work, Washington University in St. Louis, St. Louis, MO, United States of America; 2 School of Social Work, University of North Carolina, Chapel Hill, Chapel Hill, NC, United States of America; 3 Institute for Health Research and Policy, University of Illinois Chicago, Chicago, IL, United States of America; 4 Children’s Hospital Los Angeles, Los Angeles, CA, United States of America; 5 Department of Pediatrics, Keck School of Medicine, University of Southern California, Los Angeles, CA, United States of America; Public Library of Science, UNITED STATES OF AMERICA

## Abstract

Sexual minority men of color report intimate partner violence (IPV) and substance use at elevated rates compared to heterosexual peers, but little is known about how types (physical/sexual, controlling, monitoring, emotional) of perpetration and victimization are connected to types of substance use. Associations between past-6-month IPV experiences and substance use (tobacco, alcohol, cannabis, poppers, cocaine) were examined among sexual minority men (N = 414; 18–27 years). IPV victimization and perpetration were reported by 22% and 14% of the sample. Any victimization and controlling victimization were positively correlated with tobacco use, physical victimization was positively correlated with cocaine and poppers use, and monitoring victimization was negatively correlated with cannabis and poppers use. Any perpetration was positively correlated with tobacco use and binge drinking, and emotional perpetration was positively correlated with binge drinking. Understanding and addressing IPV victimization and perpetration experiences are critical for understanding risk conferred by IPV in this population.

## Introduction

Research has documented higher rates of intimate partner violence (IPV) among minority populations, including racial and ethnic minorities [1–3] and sexual minorities (i.e., lesbian, gay, bisexual, and queer-identified individuals) [3–6]. A large national study of cohabitating couples reported that incidences of IPV among Black and Hispanic couples were two times higher than White couples but did not investigate the role of sexual identity or type of IPV experienced [2]. Similarly, a critical review of IPV among sexual minority populations found that IPV rates were equal to or greater than those of heterosexual persons [4]. Minority stress theory has been posited as model for why increased health disparities, including elevated rates of IPV, are faced by minoritized populations [7–11]. Indeed, recent work has linked elevated rates of IPV among Latino sexual minority men to their experiences of race-based discrimination [12]. Given the additional minority stressors that may be experienced by individuals and groups who hold multiply minoritized identities, targeted effort must be dedicated to understanding the unique health and social needs of those most impacted [13].

Additionally, literature on IPV victimization and perpetration has shown significant associations with alcohol and other substance use [14]. For example, a meta-analytic review including 258 studies reported that alcohol and drug use were significantly associated with IPV (perpetration and victimization) with mean effect-size ranging from r = 0.18 to 0.23 [15]. This review indicated a significantly stronger correlation between drug use and victimization, compared to alcohol use and victimization. Another recent systematic review found mixed results across studies for the association between alcohol use and IPV perpetration among Black or African American men [16]. Overall, however, few studies investigate relations between IPV victimization and perpetration and reports of substance use among sexual minority men of color (e.g., Black, Latino) and there are several critical knowledge gaps.

First, empirical research has rarely documented types of IPV victimization and perpetration experiences (i.e., physical/sexual, emotional, controlling, and monitoring types of violence) among young Black and Latino sexual minority men. Among the few studies that have conducted such investigations, research relied on small samples [17], did not break down particular types of IPV [17], and considered victimization but not perpetration [3, 18, 19]. Furthermore, insufficient attention has been paid to young adulthood (under age 25) [8], a critical period when IPV experiences are most likely to onset [20].

Second, only limited research has examined how use of different substances (e.g., tobacco, alcohol, cannabis, poppers) may be associated with different types of IPV victimization and perpetration experiences among sexual minority men of color. In a study with Black same-sex couples, Wu et al. found that heavy drinking was significantly associated with IPV perpetration and victimization (lifetime and past 30 days) whereas crack-cocaine use was significantly associated with lifetime victimization and perpetration with their current partner [17]. Duncan et al. found a positive association between sexual victimization and aggregated substance use, but particular substances used were not reported [18].

### The present study

This study addresses these gaps and responds to suggestions of researchers recommending additional research on IPV among sexual minority Black and Latino men [1], particularly those recommendations suggesting consideration of various types of IPV perpetration and victimization [14]. This study documents rates of IPV victimization and perpetration—including types of IPV—among a young urban sample of Black and Latino sexual minority men and examines how different types of substance use are associated with different types of IPV victimization and perpetration experiences. In line with the limited research in this area [17, 18], we expect overall IPV victimization and perpetration to be positively associated with binge drinking, cannabis, poppers, and cocaine. Given the limited of research on *types* of IPV and substance use among sexual minority Black and Latino men, we anticipate associations of types of IPV with some substances, however we make no a priori hypotheses about which associations will be significant.

## Methods

### Participants and procedures

Data were from the Healthy Young Men’s Cohort Study (HYMS; *N* = 448) [21]. Recruitment included venue-based, social media, and referrals from participants and health clinics. Eligible individuals were (1) 16 to 24 years old at recruitment; (2) assigned male sex at birth; (3) self-identified as gay, bisexual or uncertain about their sexual identity; (4) reported a sexual encounter with a man in the previous 12 months; (5) self-identified as African American/Black, Hispanic/Latino or multi-racial/ethnic; and (6) lived in the Los Angeles area. This study used data from the fifth wave of longitudinal data collection which ran from April 2018 to September 2019 (n = 414). For each participant, this was roughly two years after their initial enrollment into the study. Notably, while data collection continued with this cohort for an additional three waves (for a total of eight waves) this was the last full wave of data collection before the COVID-19 pandemic lockdown period which started in March 2020. All participants provided written informed consent during face-to-face meetings at baseline; a waiver of parental consent/assent was obtained for participants under the age of 18 at baseline. Participant retention was high across all waves, including the fifth wave (87%-90% at waves 2–5). This study was approved by the Children’s Hospital Los Angeles Institutional Review Board.

### Measures

#### Intimate partner violence predictors

Participants completed 36 items assessing past-6-month IPV victimization and perpetration using an instrument designed for and validated with gay and bisexual men [22]. This instrument assesses four factors: physical/sexual IPV (6 items; victimization α = .69, perpetration α = .80; e.g., “Did any of your sexual partners force you to do something sexually when you did not want to?”); emotional IPV (3 items; victimization α = .67, perpetration α = .58; e.g., “Did any of your sexual partners call you fat or ugly?”); controlling IPV (4 items; victimization α = .77, perpetration α = .81; e.g., “Did any of your sexual partners prevent you from seeing your friends?”); and monitoring IPV (5 items; victimization α = .80, perpetration α = .77; e.g., “Did any of your sexual partners demand access to your cell phone?”). Each question was asked twice with adaptive wording to separately assess victimization and perpetration.

#### Substance use outcomes

Self-reported substance use was assessed in the six months prior to data collection and included tobacco, alcohol, cannabis, poppers, and cocaine use. Any past-6-month use of tobacco, cannabis, poppers, and cocaine use were considered (yes/no). Tobacco use was assessed using items adapted from the National Youth Tobacco Survey [23]; cannabis, poppers, and cocaine use was assessed using items adapted from Monitoring the Future [24]. For alcohol use, any past-6-month binge drinking was considered (yes/no) and was assessed as consuming 5 or more drinks containing any kind of alcohol within a two-hour period [25].

#### Demographic covariates

Participants self-reported a variety of demographic characteristics: race/ethnicity (Latino [referent], Black, multi-racial/ethnic); age (years); sexual identity (gay [referent], bisexual, other), HIV status (negative [referent], positive), employment status (employed [referent], not employed), and level of food security (secure [referent], insecure, hunger).

### Statistical analysis

Univariate statistics were used to describe the sample demographics and determine the amount of IPV victimization, perpetration, and substance use among the participants. Chi-square bivariate tests were conducted to determine which categorical demographic covariates should be retained in multivariate models while t-tests were conducted to examine a relationship between age and substance use in the past six months. Model assumptions were examined and met, with adjustments made for any violations. While multicollinearity was determined to not be an issue, models of any IPV and specific types of IPV were separated given that anyone reporting a specific type of IPV victimization or perpetration (e.g. monitoring IPV victimization) would automatically also be coded to have experienced “any” IPV, thus ensuring high collinearity. Linearity is not assumed for the relationship between categorical variables; however, the continuous variable of age was tested for linearity with the log-odds of each substance use outcome. Linearity was violated for only one substance use outcome, cocaine, however no adjustments were made to ease interpretation of any potential findings.

Logistic regression modeling was used to investigate associations between past-6-month IPV experiences and substance use among our sample of young sexual minority participants of color assigned male sex at birth. Two models were fit for each substance use outcome: one examined any IPV victimization or perpetration and another examined IPV victimization and perpetration by type (physical/sexual, controlling, monitoring, emotional). To analyze sensitivity of the substance use outcomes by demographics, we compared models including all demographic covariates (race/ethnicity, age, sexual identity, HIV status, employment status, and level of food security) to models with just covariates found to be significant during bivariate testing. Due to the exploratory nature of this investigation, to avoid making Type II errors (false negatives), no corrections were made for multiple testing. All modeling was completed using Stata/MP 17 [26].

## Results

Descriptive analyses (Table 1) indicated that 22% (n = 92) of participants reported any IPV victimization and 14% (n = 59) reported any IPV perpetration in the previous six months. Physical/sexual IPV victimization (n = 51; 12% of sample, 55% of victimized) was the most common type of victimization reported, followed by emotional IPV (n = 46; 11%, 50%), monitoring IPV (n = 38; 9%, 41%), and controlling IPV (n = 23; 6%, 25%). In contrast, monitoring IPV perpetration (n = 38; 9% of sample, 64% of perpetrators) was the most common type of perpetration reported, followed by emotional IPV (n = 26; 6%, 44%), physical/sexual IPV (n = 25; 6%, 42%), and controlling IPV (n = 7; 2%, 12%).

**Table 1:** Descriptive statistics, IPV exposure, and substance use among the Healthy Young Men’s Cohort, wave 5.

Variable	n or M	% or SD
Age (range 18–27 years) (n = 414)	24.06	2.01
Sexual Identity (n = 412)*		
Gay	341	83%
Bisexual	45	11%
Another identity	26	6%
Race/Ethnicity (n = 414)		
Black or African American	87	21%
Latino/e or Hispanic	244	59%
Black and Latino/e or Hispanic	83	20%
HIV Status (n = 414)		
Negative	349	84%
Positive	65	16%
Employment Status (n = 406)*		
Employed	326	80%
Unemployed	80	20%
Food Security Category (n = 414)		
Food Secure	285	69%
Food insecure	77	19%
Experiences of hunger	52	13%
IPV Exposure (past 6 months) (n = 414)		
Any IPV victimization	92	22%
Physical/Sexual IPV victimization	51	12%
Monitoring IPV victimization	35	8%
Controlling IPV victimization	23	6%
Emotional IPV victimization	46	11%
Any IPV perpetration	59	14%
Physical/Sexual IPV perpetration	25	6%
Monitoring IPV perpetration	38	9%
Controlling IPV perpetration	7	2%
Emotional IPV perpetration	26	6%
Substance use (past 6 months)		
Tobacco (n = 410)*	161	39%
Binge alcohol (5+ drinks) (n = 414)	192	46%
Cannabis (non-prescription) (n = 414)	287	69%
Cocaine (n = 414)	74	18%
Poppers (n = 414)	108	26%

*Missing due to participant decline/refusal.

Multivariable logistic regression analyses demonstrated a significant positive association between any IPV victimization and tobacco use (aOR 1.92, 95% CI: 1.10–3.36) after controlling for significant demographic covariates. Considering the specific types of IPV victimization, physical/sexual IPV was positively associated with cocaine use (aOR 2.87, 95% CI: 1.25–6.58) and poppers use (aOR 2.99, 95% CI: 1.35–6.60) and controlling IPV was significantly positively associated with tobacco use (aOR 3.28, 95% CI: 1.07–10.02). In comparison, monitoring IPV victimization was negatively associated with binge drinking alcohol (aOR 0.20, 95% CI: 0.06–0.64) and poppers use (aOR 0.18, 95% CI: 0.05–0.66). There were no significant associations for emotional IPV victimization.

In addition, multivariable logistic regression analyses (Table 2) demonstrated a significant positive association between any IPV perpetration and binge drinking alcohol (aOR 2.19, 95% CI: 1.13–4.24) after controlling for significant demographic covariates. Considering the specific types of IPV perpetration, binge drinking alcohol was positively associated with monitoring IPV (aOR 2.75, 95% CI: 1.07–7.03) and emotional IPV (aOR 4.27, 95% CI: 1.46–12.51). In comparison, there were no significant associations for physical/sexual, or controlling.

**Table 2:** Odds of substance use by IPV exposure.

Outcome of interest:	Tobacco		Binge Alcohol		Cannabis		Cocaine		Poppers	
Variable	aOR	95% CI	aOR	95% CI	aOR	95% CI	aOR	95% CI	aOR	95% CI
Any IPV exposure										
Any IPV victimization	**1.92**	**(1.10–3.36)**	0.86	(0.50–1.49)	1.35	(0.74–2.49)	1.7	(0.88–3.27)	1.62	(0.89–2.94)
Any IPV perpetration	1.21	(0.63–2.33)	**2.19**	**(1.13–4.24)**	0.77	(0.38–1.56)	1.1	(0.51–2.41)	1.19	(0.59–2.40)
Covariates										
Unemployed	**1.67**	**(1.01–2.77)**	**‐‐**	**‐‐**	**‐‐**	**‐‐**	**‐‐**	**‐‐**	**‐‐**	**‐‐**
HIV-positive serostatus	**‐‐**	**‐‐**	**‐‐**	**‐‐**	**0.58**	**(0.33–1.00)**	**‐‐**	**‐‐**	**‐‐**	**‐‐**
Food insecure [Ref: Food secure]	**‐‐**	**‐‐**	**‐‐**	**‐‐**	**‐‐**	**‐‐**	**‐‐**	**‐‐**	1.42	(0.80–2.50)
Experienced hunger [Ref: Food secure]	**‐‐**	**‐‐**	**‐‐**	**‐‐**	**‐‐**	**‐‐**	**‐‐**	**‐‐**	**2.32**	**(1.24–4.35)**
**Any IPV exposure model fit:**	**X**^**2**^_**(3)**_ **= 14.94, *p* = 0.002**		**X**^**2**^_**(2)**_ **= 6.23, *p* = 0.044**		**X**^**2**^_(3)_ = 5.14, *p* = 0.162		**X**^**2**^_(2)_ = 3.91, *p* = 0.142		**X**^**2**^_**(4)**_ **= 14.12, *p* = 0.007**	
**Outcome of interest:**	**Tobacco**		**Binge Alcohol**		**Cannabis**		**Cocaine**		**Poppers**	
**Variable**	**aOR**	**95% CI**	**aOR**	**95% CI**	**aOR**	**95% CI**	**aOR**	**95% CI**	**aOR**	**95% CI**
IPV victimization types										
Physical/Sexual IPV victimization	1.61	(0.75–3.46)	1.94	(0.89–4.25)	1.69	(0.71–4.05)	**2.87**	**(1.25–6.58)**	**2.99**	**(1.35–6.60)**
Monitoring IPV victimization	0.92	(0.34–2.48)	**0.20**	**(0.06–0.64)**	0.36	(0.12–1.03)	0.41	(0.12–1.42)	**0.18**	**(0.05–0.66)**
Controlling IPV victimization	**3.28**	**(1.07–10.02)**	1.80	(0.59–5.47)	2.48	(0.71–8.68)	1.48	(0.41–5.35)	2.00	(0.62–6.45)
Emotional IPV victimization	0.69	(0.31–1.52)	0.73	(0.34–1.58)	0.85	(0.38–1.86)	0.84	(0.33–2.12)	1.19	(0.53–2.67)
IPV perpetration types										
Physical/Sexual IPV perpetration	0.67	(0.21–2.16)	1.06	(0.32–3.49)	0.71	(0.21–2.44)	0.94	(0.26–3.41)	1.11	(0.33–3.76)
Monitoring IPV perpetration	1.82	(0.76–4.40)	**2.75**	**(1.07–7.03)**	1.22	(0.48–3.12)	1.78	(0.64–4.95)	1.45	(0.54–3.90)
Controlling IPV perpetration	1.02	(0.14–7.36)	1.03	(0.13–8.13)	0.8	(0.11–6.10)	0.84	(0.10–7.08)	2.47	(0.3–20.35)
Emotional IPV perpetration	1.43	(0.55–3.75)	**4.27**	**(1.46–12.51)**	1.54	(0.53–4.44)	1.11	(0.35–3.51)	1.04	(0.35–3.10)
Covariates										
Unemployed	**1.77**	**(1.06–2.95)**	**‐‐**	**‐‐**	**‐‐**	**‐‐**	**‐‐**	**‐‐**	**‐‐**	**‐‐**
HIV-positive serostatus	**‐‐**	**‐‐**	**‐‐**	**‐‐**	**0.55**	**(0.31–0.96)**	**‐‐**	**‐‐**	**‐‐**	**‐‐**
Food insecure [Ref: Food secure]	**‐‐**	**‐‐**	**‐‐**	**‐‐**	**‐‐**	**‐‐**	**‐‐**	**‐‐**	1.43	(0.8–2.58)
Experienced hunger [Ref: Food secure]	**‐‐**	**‐‐**	**‐‐**	**‐‐**	**‐‐**	**‐‐**	**‐‐**	**‐‐**	**2.46**	**(1.28–4.71)**
**IPV exposure subtypes model fit:**	**X**^**2**^_**(9)**_ **= 19.18, *p* = 0.024**		**X**^**2**^_**(8)**_ **= 20.15, *p* = 0.010**		**X**^**2**^_(9)_ = 9.56, *p* = 0.387		**X**^**2**^_(8)_ = 9.10, *p* = 0.334		**X**^**2**^_**(10)**_ **= 26.08, *p* = 0.004**	

## Discussion

These findings add to the literature on types of IPV victimization and perpetration among young Black and Latino sexual minority men. Notably, although physical/sexual IPV was the most common among those reporting victimization, monitoring IPV was the most common among those reporting perpetration. Consistent with our hypotheses about general associations between IPV and substance use, any IPV victimization was associated with increased likelihood of tobacco use and any IPV perpetration was associated with increased likelihood of binge drinking. However, our results also suggested nuanced associations between types of victimization and perpetration and types of substances. Physical/sexual IPV victimization was linked to stimulating, social substances (poppers, cocaine), which are also linked to increased risky sexual behaviors [27, 28]. Although we were unable to examine reasons and processes underlying participants’ use of stimulating, social drugs, this finding could stem from use to cope with IPV experiences or use under coercion manifesting from IPV victimization. In contrast, increased monitoring IPV victimization was linked to lower likelihoods of cannabis and poppers use, substance that are also used socially. This finding could stem from social restriction as an extension of behavior-monitoring mechanisms and/or alienation of from social circles. Further, emotional IPV perpetration was linked to binge drinking. This is consistent with meta-analytic results on risk factors for IPV in same-sex relationships, where alcohol misuse was reported as a significant risk for IPV perpetration [29]. Critically, despite elevated rates of self-reported IPV victimization and perpetration over recent years, interventions addressing IPV among sexual minority populations are sorely lacking.

Results presented here must be considered alongside study limitations. Data analyzed stem from a single study wave thus temporality of IPV and substance use cannot be determined. As additional IPV data are collected in HYMS, future work will be better positioned to unpack temporality. Notably, the reliability of the emotional IPV victimization subscale was in an unacceptable range (< .60) which may have led to the lack of findings in that area. Additionally, caution should be taken when interpreting the results of the models of cannabis and cocaine use, as the overall models in those cases were not statistically significant. The HYMS questionnaire did not assess additional information about specific IPV experiences (e.g., context). Behaviors captured as violence may have been consensual in some instances. Similarly, it is possible that a partner was monitoring communications at the behest of the other. Interestingly, fewer participants reported IPV perpetration than victimization, which could be related to social desirability bias. Questions about IPV and substance use were self-reported using a computer-based survey, not through interviewer led assessment, however, so this bias should be minimized. Future studies interested in IPV should collect contextual information and follow up to ensure experiences are captured and interpreted correctly. While assigned male sex at birth was an inclusion criterion for this study, gender was not. Our findings should be interpreted with the understanding that while 95% of the sample identified their gender as male in wave 5, the remaining 5% did not. Additionally, gender was fluid throughout the study for some participants. Finally, these results may not be generalizable outside of young sexual minority people of color assigned male sex at birth in Los Angeles.

These findings expand on existing literature linking IPV and substance use generally. Understanding specificity of IPV experiences and their relations to substance use provides nuanced information for interventions. Namely, programs and services targeting IPV victims or perpetrators and/or those who misuse substances will be exponentially more successful if they address the specific needs of those they engage. To adapt and update existing interventions developed for other populations (e.g., heterosexual women) we must understand the unique experiences of young sexual minority people of color assigned male sex at birth. Ultimately, IPV and substance misuse are preventable, but equity will remain elusive until impacted communities are understood and are engaged in developing and testing the tools meant to improve their lives.

## Supporting information

S1 Data (CSV)
